# Validation of method for faecal sampling in cats and dogs for faecal microbiome analysis

**DOI:** 10.1186/s12917-023-03842-7

**Published:** 2023-12-16

**Authors:** Xavier Langon

**Affiliations:** Royal Canin Sas, 650 avenue de la Petite Camargue, AIMARGUES Cedex, CS 10309, 30470 France

**Keywords:** Cat, Dog, Microbiome, Gastrointestinal, Faeces, Storage, Temperature

## Abstract

**Background:**

Reproducible and reliable studies of cat and dog faecal microbiomes are dependent on many methodology-based variables including how the faecal stools are sampled and stored prior to processing. The current study aimed to establish an appropriate method for sampling and storing faecal stools from cats and dogs which may also be applied to privately-owned pets. The approach investigated the effects of storing faeces for up to 12 h at room temperature and sampling from various locations within the stool in terms of microbial diversity, relative taxa abundances and DNA yield. Faeces were collected from 10 healthy cats and 10 healthy dogs and stored at room temperature (20 °C). Samples were taken from various locations within the stool (the first emitted part (i), the middle (ii) and the last emitted end (iii), at either surface or core) at 0, 0.5, 1, 2, 3, 6 and 12 h, stabilised and stored at -80 °C. DNA was extracted from all samples, using Illumina NovaSeq.

**Results:**

Faecal bacterial composition of dogs and cats shown no statistically significant differences in alpha diversity. Bacteroidetes, Firmicutes, Proteobacteria and Actinobacteria were the most prevalent phyla. Cat and dog samples were characterized by a dominance of Prevotella, and a lack of Fusobacterium in feline stools. Room temperature storage of cat and dog faecal samples generally had no significant effect on alpha diversity, relative taxa abundance or DNA yield for up to 12 h. Sampling from regions i, ii or iii of the stool at the surface or core did not significantly influence the outcome. However, surface cat faecal samples stored at room temperature for 12 h showed a significant increase in two measures of alpha diversity and there was a tendency for a similar effect in dogs. When comparing samples with beta diversity measures, it appeared that for dog and cat samples, individual effect has the strongest impact on the observed microbial diversity (R2 0.64 and 0.88), whereas sampling time, depth and horizontal locations significantly affected the microbial diversity but with less impact.

**Conclusion:**

Cat and dog faeces were stable at room temperature for up to 12 h, with no significant changes in alpha diversity, relative taxa abundance and DNA concentration. Beta diversity analysis demonstrated that despite an impact of the sampling storing time and the surface of the sampling, we preserved the identity of the microbial structure linked to the individual. Finally, the data suggest that faecal stools stored for > 6 h at room temperature should be sampled at the core, not the surface.

**Supplementary Information:**

The online version contains supplementary material available at 10.1186/s12917-023-03842-7.

## Background

Billions of microorganisms, in particular bacteria, inhabit the gastrointestinal tract of cats and dogs where they play a key role in sustaining host health [1]. Studies continue to use increasingly sophisticated molecular techniques to characterise the cat and dog gastrointestinal microbiome and to understand how it is influenced by factors such as age, diet, medication, and disease state [2–8]. It is recognised that the microbial community alters along the various sections of the gastrointestinal tract, with faecal samples offering an accepted and practical method of gut microbiome investigation [9, 10].

Studies of the cat and dog faecal microbiome have largely involved relatively limited numbers of research colony animals and there is a need for investigation in larger cohorts that represent the broader population. Sample collection, handling and storage are critical steps that can alter the accuracy and reproducibility of downstream bacterial DNA analysis therefore it is critical to standardise these factors before embarking on such studies.

One of the issues associated with faecal analysis in privately-owned cats and dogs is the ability to obtain a fresh sample, especially from cats which may defaecate in the litter box overnight. Human studies have shown faeces stored for 48 h at ambient temperature show changes in faecal microbiota composition [11, 12]. Reports describing the effect of companion animal faecal sample storage at ambient temperature on the bacterial population are scarce and tend to study much longer storage intervals. One such study found that storage of cat faeces for up to four days at ambient temperature had no apparent effect on the bacterial population [13]. A study on dog faeces found that storage of stabilised samples at ambient temperature for 14d did not affect alpha or beta microbial diversity, although unstabilised samples stored under the same conditions showed higher alpha diversity and altered relative abundance of dominant bacterial phyla, highlighting the importance of a stabilisation system [14].

There is evidence in humans to suggest that rectal swabs show bacterial composition differences when compared with rectal stool samples, with differing oxygen gradients in mucosal and luminal samples reported to influence gut microbiota data [15, 16]. However, there are no reports comparing the gut microbiota at different locations of the faecal stool. These findings suggest that the influence of horizontal sampling location on the outcome should also be considered as well as the depth of sampling (surface versus core). Taken together, the above evidence supports the need to validate a standardised approach to faecal sampling and processing that will be appropriate for gut microbiome analysis within the research environment, and which can also be successfully applied to privately-owned pets.

The current study aims to validate a cat and dog faecal sampling and processing method in a group of research colony animals to inform on a suitable approach for further application onsite and in privately-owned animals. In particular, the study aims to understand the effect of storage for up to 12 h at room temperature on bacterial DNA concentration, microbial diversity, and relative abundance of taxa in cat and dog faecal samples. A maximum time of 12 h was selected as this corresponds with the maximum expected delay between a client-owned animal defaecating at home and the owner collecting and stabilising the sample. The study also aims to clarify how the above measures are influenced by horizontal and depth sampling location within the faecal stool by comparing samples from the first emitted part (i), the middle (ii) and last emitted end (iii), taken from the surface (S) or core (C). In addition, two stabilisation solutions are compared to determine their influence on the measures described.

## Results

A total of 263 samples taken from 20 stools from 10 dogs and 10 cats were extracted and sequenced. Two samples were missing in total due to insufficient sample size, meaning that timepoint two hours (T2) for cats number 7 (C7) and number 8 (C8) were omitted. All faeces produced were of acceptable consistency, graded 2–3 for cats and 2.5-3 for dogs. Total sequencing reads comprised over 90% high quality reads.

### Effect of animal species on faecal metagenomic profiles and diversity

Samples were clustered by individuals to produce a unique faecal microbial signature for almost all animals. One dog sample (dog number 4 (D4) individual at time 720 (T720) on surface) was very dissimilar to all other dogs, with only 14 bacterial species detected so was removed from all other statistical comparisons (supplementary Fig. 1).

**Fig. 1:**
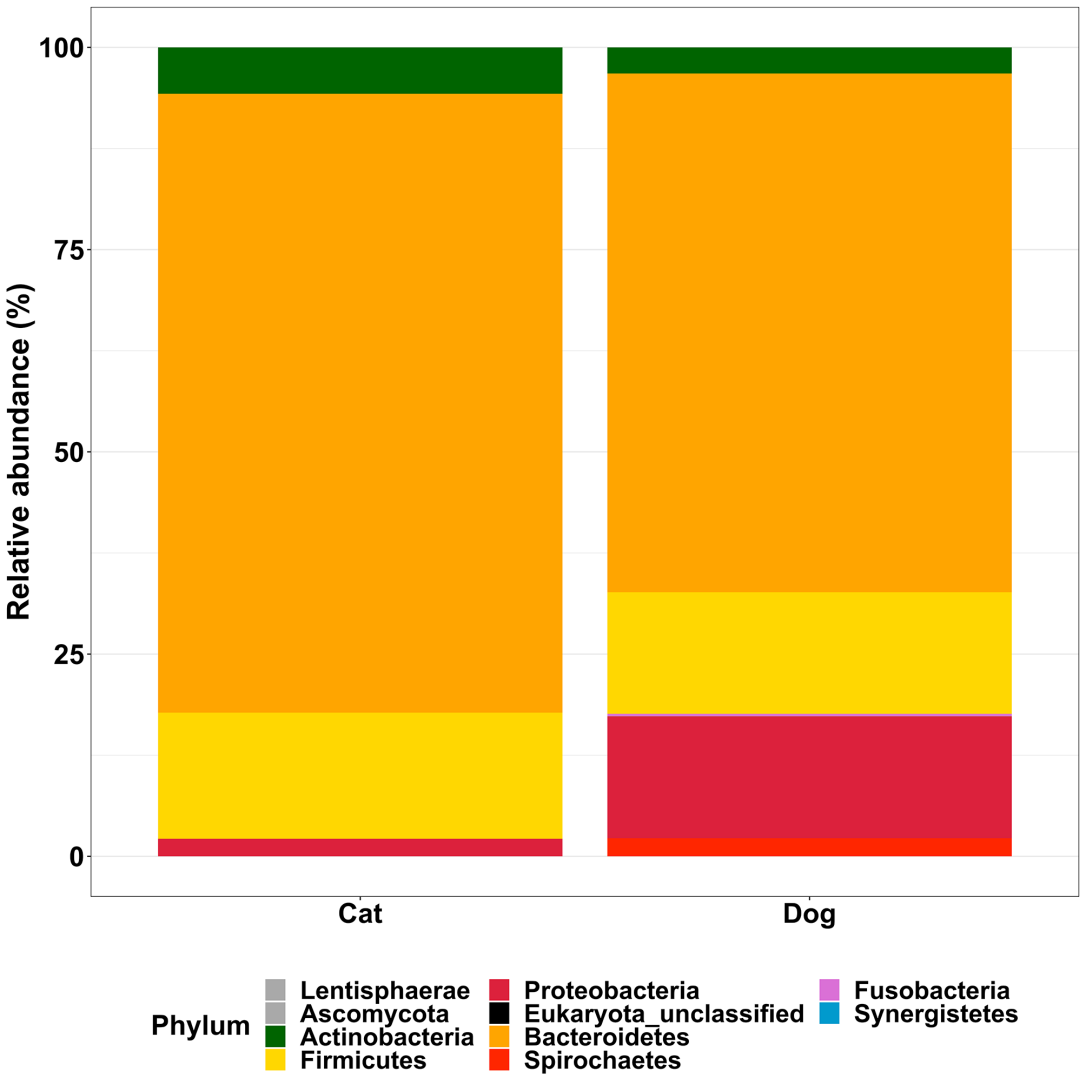
Relative abundance of predominant phyla in faecal samples from 10 healthy cats and 10 healthy dogs

Overall, consistent differences were found in the faecal bacterial composition of dogs when compared with cats. At the phylum level, all cat faeces were dominated by Bacteroidetes, followed by Firmicutes/Actinobacteria and Proteobacteria (Fig. 1; Table 1). Spirochaetes and Fusobacteria were absent in cats.

All dogs, with the exception of two individuals, showed a high relative abundance of Bacteroidetes, followed by Firmicutes, Proteobacteria and Actinobacteria (Fig. 1; Table 1). Spirochaetes and Fusobacteria were also present in some dogs with overall prevalence of 38.1% and 10.4% respectively. Bacteroidetes, Actinobacteria and Firmicutes were the only phyla showing 100% prevalence in both cats and dogs.

**Table 1:** Prevalence and relative abundance of bacterial phyla in dog and cat faecal samples selected according to criteria (Ozyme samples, at T0, Core and Middle samples)

Phylum	Animal species	Prevalence (%)	Mean relative abundance (%) (SD)
Actinobacteria	Cat	100	5.7 (8.6)
	Dog	100	3.2 (3.4)
Bacteroidetes	Cat	100	76.5 (16.0)
	Dog	100	64.1 (22.4)
Firmicutes	Cat	100	15.6 (8.3)
	Dog	100	15.0 (15.2)
Fusobacteria	Cat	0.0	0.0
	Dog	10.4	0.3 (1.2)
Proteobacteria	Cat	96.1	2.2 (5.99)
	Dog	97.8	15.1 (15.1)
Spirochaetes	Cat	0.0	0.0
	Dog	38.1	2.2 (5.6)

Values for > 10% prevalence shown, based on faecal samples from ten healthy cats and ten healthy dogs

**Fig. 2:**
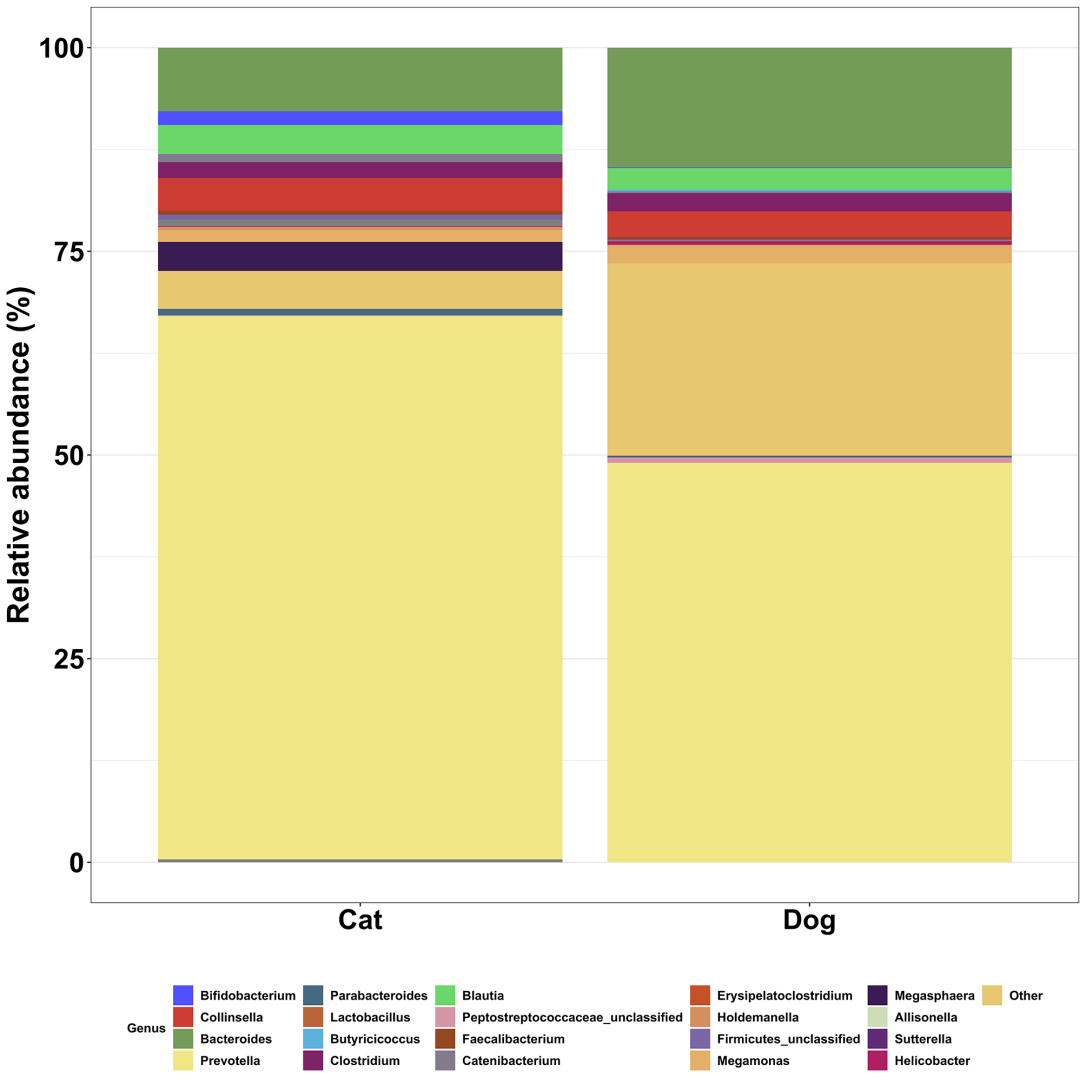
Relative abundance of predominant genera in faecal samples from 10 healthy cats and 10 healthy dogs

When considering relative abundance at the genus level, Prevotella was dominant across all cats, making up 66.7% of classified reads (Fig. 2; Table 2). Although still the most dominant genus in dogs, Prevotella generally made up 49.0% of classified reads (Fig. 2; Table 3). Prevalence of Prevotella was 100% for both cats and dogs. Bacteroides showed 100% prevalence in cats and dogs and represented the next most abundant genus for both species (means 14.7 and 7.8% respectively) followed by an unclassified Proteobacteria in dogs (mean abundance 7.1%) and Collinsella in cats (mean abundance 4.0%). Other major genus differences between cats and dogs include a 1.7% mean abundance of Bifidobacteria in cats (< 0.1% in dogs), 5.4% mean abundance of Escherichia in dogs (< 0.01% in cats), 3.6% Streptococcus in dogs (< 0.001% in cats) and 3.6% mean abundance of Megasphaera in cats yet an absence in dogs.

**Table 2:** Prevalence and relative abundance of bacterial genera in dog and cat faecal selected according to criteria (Ozyme samples, at T = 0, Core and Middle samples)

Genus	Animal species	Prevalence (%)	Mean relative abundance (%) (SD)
Bacteroides	Cat	100.0	7.8 (6.12)
	Dog	100.0	14.7 (12.7)
Bifidobacteria	Cat	88.3	1.7 (3.3)
	Dog	35.8	< 0.1
Blautia	Cat	100.0	3.6 (3.0)
	Dog	100.0	2.6 (4.05)
Collinsella	Cat	100.0	4.0 (7.1)
	Dog	100.0	3.2 (3.5)
Escherichia	Cat	7.0	< 0.1
	Dog	76.1	5.4 (11.3)
Megasphaera	Cat	100.0	3.6 (4.2)
	Dog	0.0	0.0
Prevotella	Cat	100.0	66.7 (17.3)
	Dog	100.0	49.0 (28.4)
Proteobacteria	Cat	0.0	0.0
	Dog	38.8	7.1 (12.1)
Streptococcus	Cat	2.3	< 0.1
	Dog	49.2	3.6 (7.5)

Top five most abundant genera for each species shown, based on faecal samples from ten healthy cats and ten healthy dogs

**Fig. 3:**
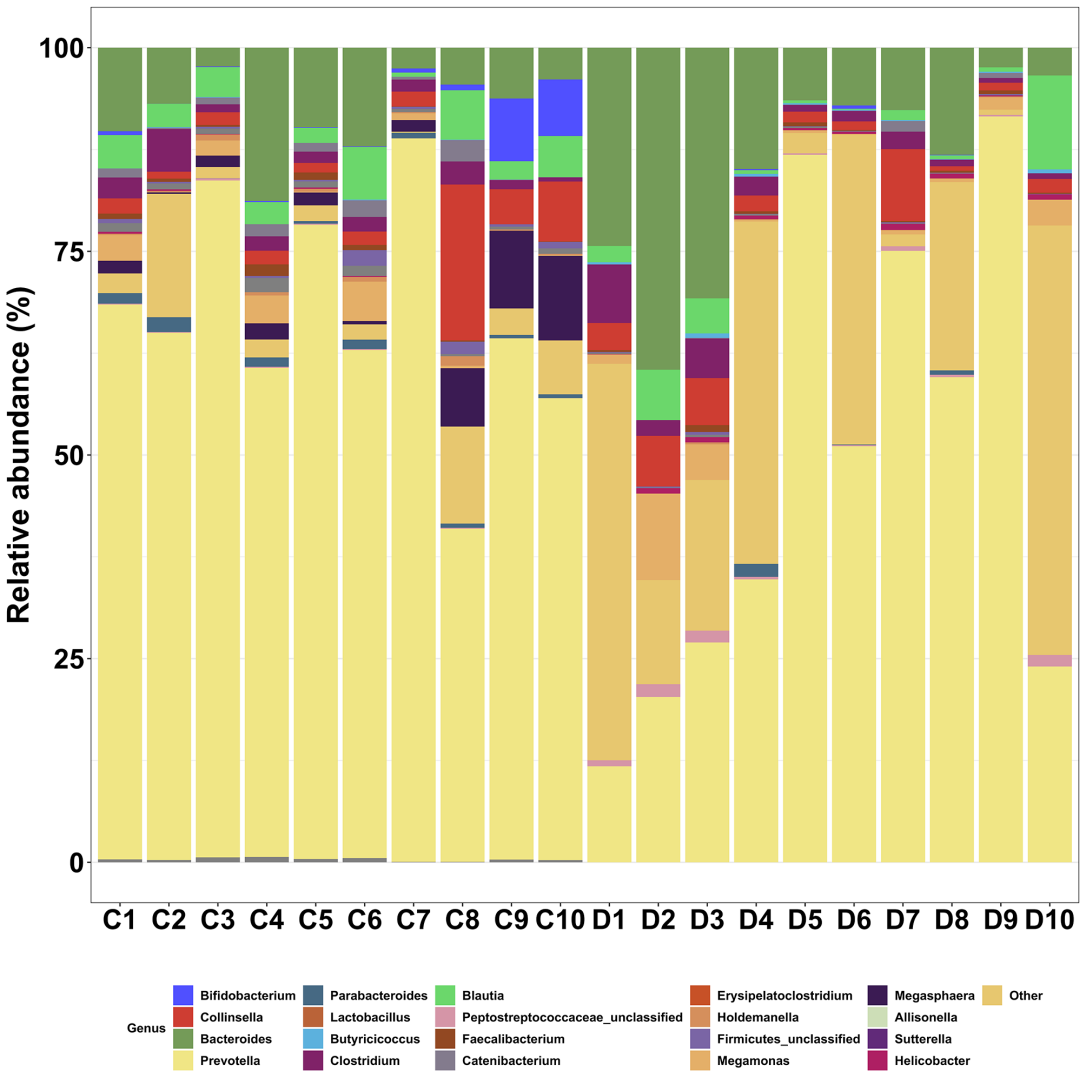
Relative abundance of predominant genera in faecal samples from 10 healthy cats (**C**) and 10 healthy dogs (**D**), by individual

Comparisons of relative abundance by genus across individuals demonstrated individual variability (Fig. 3).

Species detected in almost all cats but not dogs were: *Blautia wexlerae, Collinsella aerofaciens, Flavonifractor plautii, Megasphaera elsdenii, Ruminococcaceae bacterium D16, Fusicatenibacter saccharivorans, Bifidobacterium pullorum*. *Gemmiger* sp An194 and *Helicobacter bilis* were detected in all dogs and not in cats. When relative abundances of species with high prevalence (> 60% in both cats and dogs) were compared across cat and dog, five species were significantly different (adjusted *p* < 0.05) and 4 highly significantly different (adjusted *p* < 0.01; Table 3).

**Table 3:** Most prevalent bacterial species in cat and dog faeces samples selected according to the folloeing criteria: Ozyme tube samples, at T = 0, core and middle samples

Bacterial species	Adjusted *p* value	Significance level	Mean relative abundance cat (%)(SD)	Mean relative abundance dog (%) (SD)
*Butyricicoccus pullicaecorum*	0.03	*	0.03 (0.05)	0.22 (0.2)
*Catenibacterium mitsuokai*	0.02	*	0.97 (1.04)	0.22 (0.5)
*Clostridium hiranonis*	0.008	**	0.10 (0.1)	0.67 (0.8)
*Collinsella intestinalis*	0.003	**	0.18 (0.22)	3.06 (3.44)
*Collinsella stercoris*	0.003	**	2.16 (3.42)	0.09 (0.08)
*Firmicutes bacterium* CAG424	0.005	**	0.34 (0.0)	0.05 (0)
*Firmicutes bacterium* CAG646	0.03	*	0.21 (0.0)	0.05 (0)
*Holdemanella biformis*	0.01	*	0.39 (0.43)	0.06 (0)
*Prevotella copri*	0.02	*	65.1 (16.7)	29.5 (27.3)

Significant differences in relative abundance between cat and dog core faeces sample taken at timepoint T0. Adjusted *p* values refer to Benjamini-Hochberg corrected Wilcoxon rank sum test comparisons

**Fig. 4:**
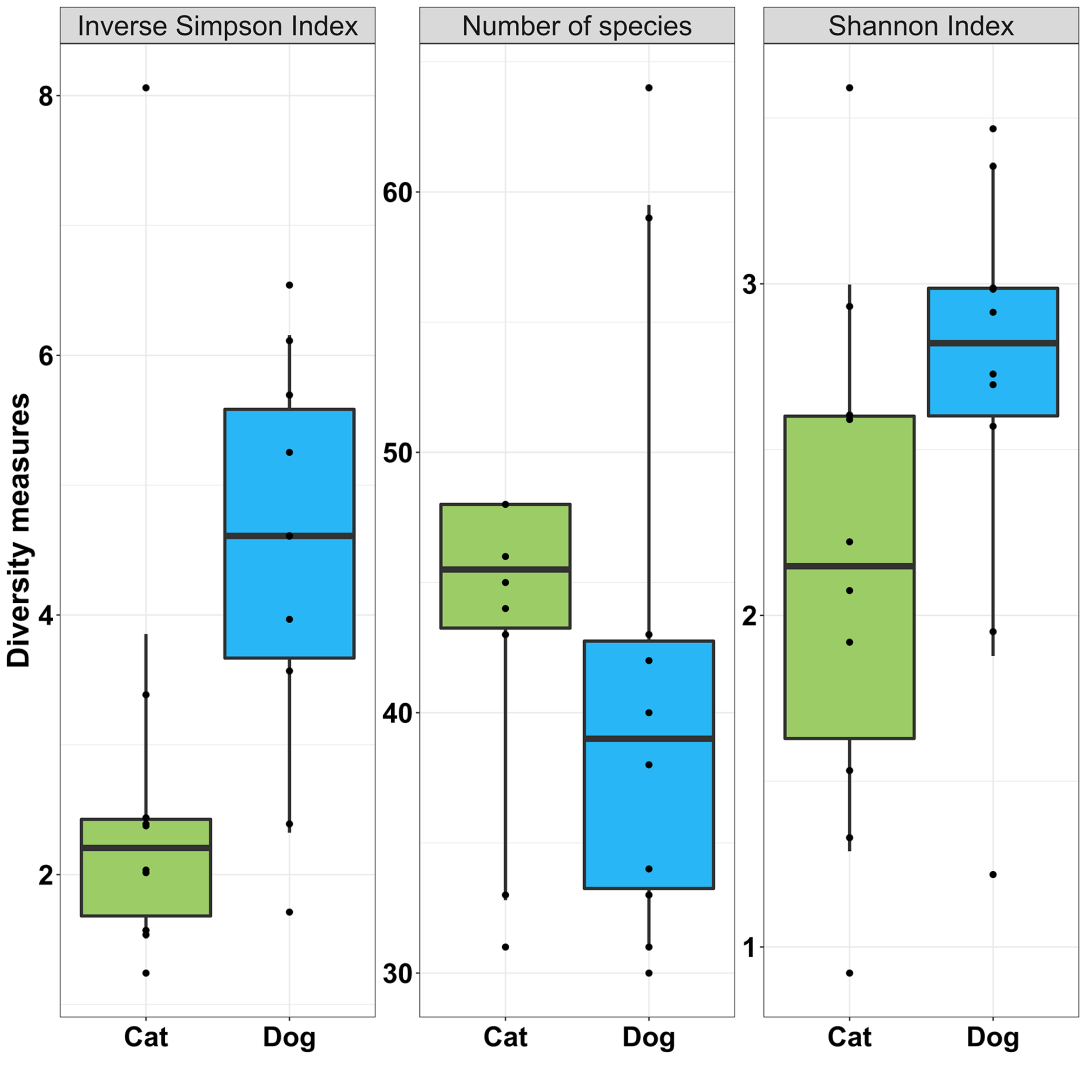
Cat and dog faecal microbial diversity according to three methods. Samples were taken at T0 from region ii of faeces from 10 healthy cats and 10 healthy dogs

Alpha diversity was compared between cats and dogs using a standardised T0 sample taken from the first emitted part (i) core region of the faecal stool. Similar numbers of bacterial species were identified in cats and dogs, with a mean of 43.4 ± 6.3 bacterial species in cats (median 45.5) and 41.4 ± 11.5 in dogs (median 39.0; adjusted *p* = 0.20; Fig. 4).

Although not statistically significant, mean alpha diversity as measured by Inverse Simpson Index tended to be higher in dogs (4.4 ± 1.6) compared with cats (2.7 ± 2.0; adjusted *p* = 0.05). Similarly, mean Shannon Index diversity tended to be higher in dogs compared with cats (adjusted *p* = 0.18). Overall, there were no statistically significant differences in alpha diversity between dogs and cats although there was a pattern towards slightly higher diversity in dogs.

### Effect of experimental variables on microbial diversity and relative taxa abundance

**Fig. 5:**
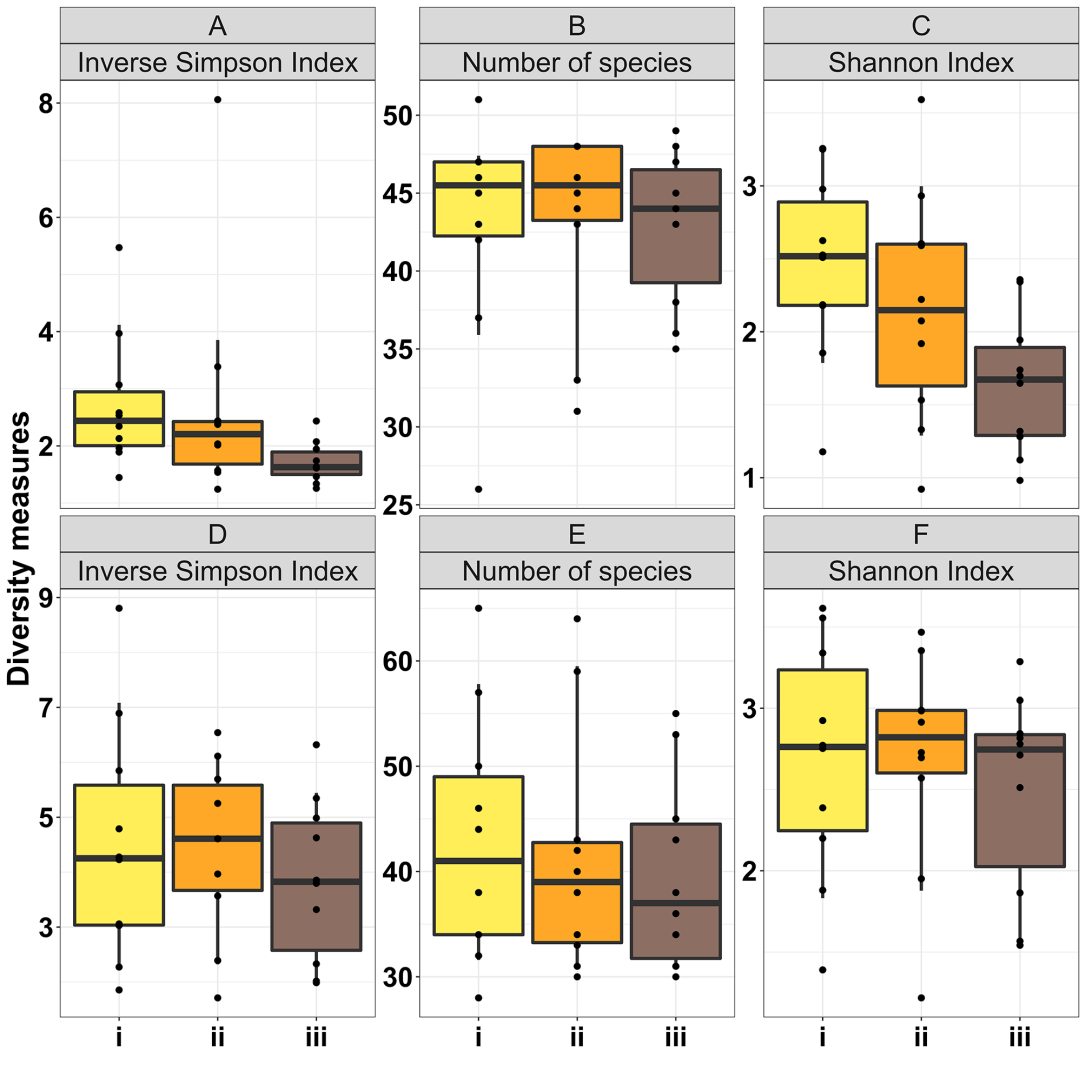
Comparisons of microbial diversity between regions i, ii and iii in healthy cat (**A-C**) and healthy dog (**D-F**) stool samples at T0

In standardised samples taken at T0 (core), there was no significant effect of the three horizontal sampling locations on microbial diversity in cats or dogs, as determined by Inverse Simpson Index (cat adjusted *p* = 0.06; dog adjusted *p* = 0.81), number of species (cat adjusted *p* = 0.85; dog adjusted *p* = 0.81) and Shannon Index (cat adjusted *p* = 0.06; dog adjusted *p* = 0.81) (Fig. 5).

**Fig. 6:**
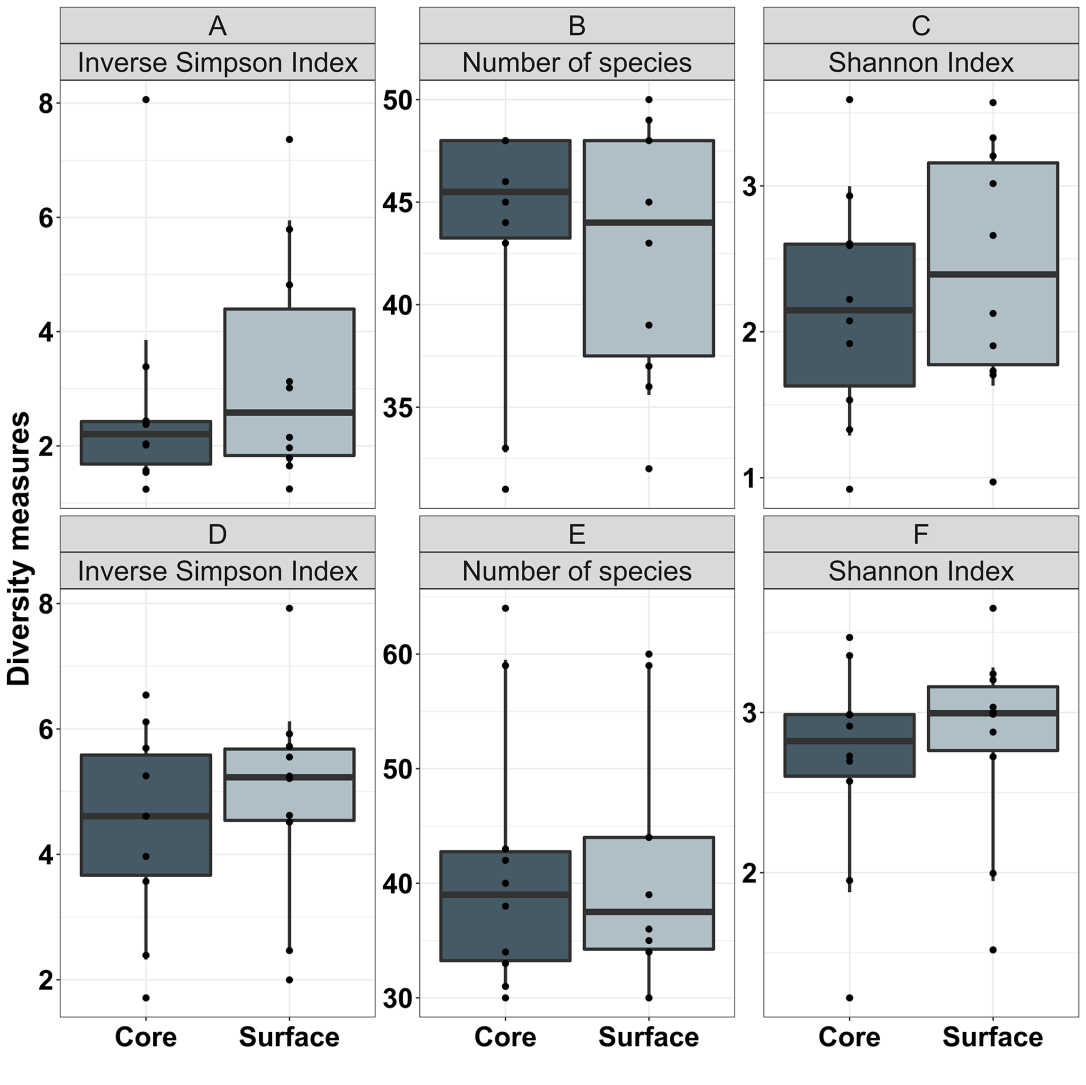
Comparisons of microbial diversity between core and surface samples from healthy cat (**A-C**) and healthy dog (**D-F**) faecal stools stored at room temperature for 12 h

Comparisons of core and surface samples from region ii at T0 showed no effect of sampling depth on microbial diversity in cats and dogs (adjusted *p* values all > 0.05). At T12, cat surface samples from region iii showed increased mean alpha diversity when compared with core samples from the same region (Shannon Index: 2.3 ± 0.7 at surface and 1.8 ± 0.7 in core, adjusted *p* = 0.018; Inverse Simpson Index: 2.7 ± 1.6 at surface and 2.3 ± 1.8 in core, adjusted *p* = 0.048), but this was not true for diversity measured by number of species (adjusted *p* = 0.06; Fig. 6). There was increased alpha diversity as measured by Shannon Index for dogs in region iii surface (2.6 ± 0.7) compared with region iii core samples (2.4 ± 0.7) at T12 (adjusted *p* = 0.027) but not according to Inverse Simpson Index or number of species (adjusted *p* values 0.09 and 0.37 respectively; Fig. 6).

Storage time of samples at room temperature did not influence alpha diversity in cat or dog samples, according to evaluation of the impact of time on diversity using the Kruskal-Wallis test, with adjusted *p*-values (for cats, adjusted *p* for all measures = 0.74, and for dogs = 1.00).

There were no differences in within-dog diversity when comparing the two stabilisation buffers (for all diversity measures, adjusted *p* = 0.5).

**Fig. 7:**
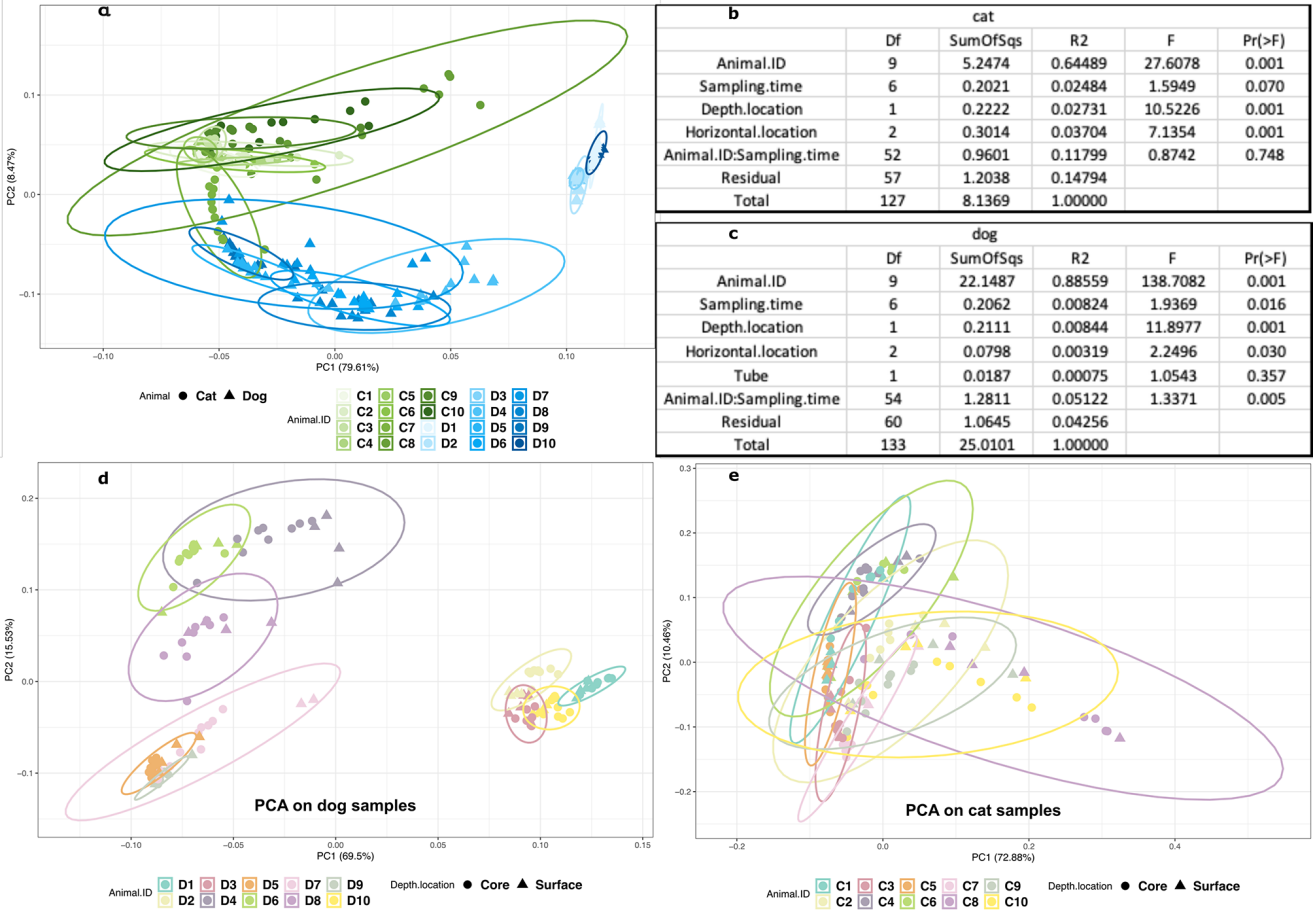
Beta diversity representation with Principal Component Analysis (PCA). **A**: PCA representation with all samples from the study; **B**: PERMANOVA results for the cat samples; **C**: PERMANOVA results for the dog samples; **D**: PCA representation with dog samples; **E** PCA representation with cat samples

Beta diversity results are presented in Fig. 7. Animal individuality is the most important factor observed that impacts the diversity (R2 of 0.64 and 0.88 for cat and dog respectively, and a probability F Prf < 0.001 in PERMANOVA results Fig. 7b and c). For the cat samples, depth and horizontal locations are also significant statistically with a probability F < 0.001 for both, but the contribution measured is less important (R2 of 0.027 and 0.037). Time sampling does not affect sample diversity for cat using beta analysis (Prf = 0.07).

For dog samples, depth location is the second most important factor to impact the sample diversity (Prf = 0.001), but the measured effect is lower than indivual effect, (R2 of 0.008). The sampling time location is also significant if we defined the threshold to 0.05. (Prf = 0.016). The importance of the effect is equal to the depth location (R2 = 0.08). Type of stabilization buffer did not have any observed effect on the sample diversity (PERMANOVA *p* value > 0.3), if there is any biases associated to the buffer we observed the same trends between the 2 tested buffers.

### Effect of experimental variables on DNA concentration in samples

**Fig. 8:**
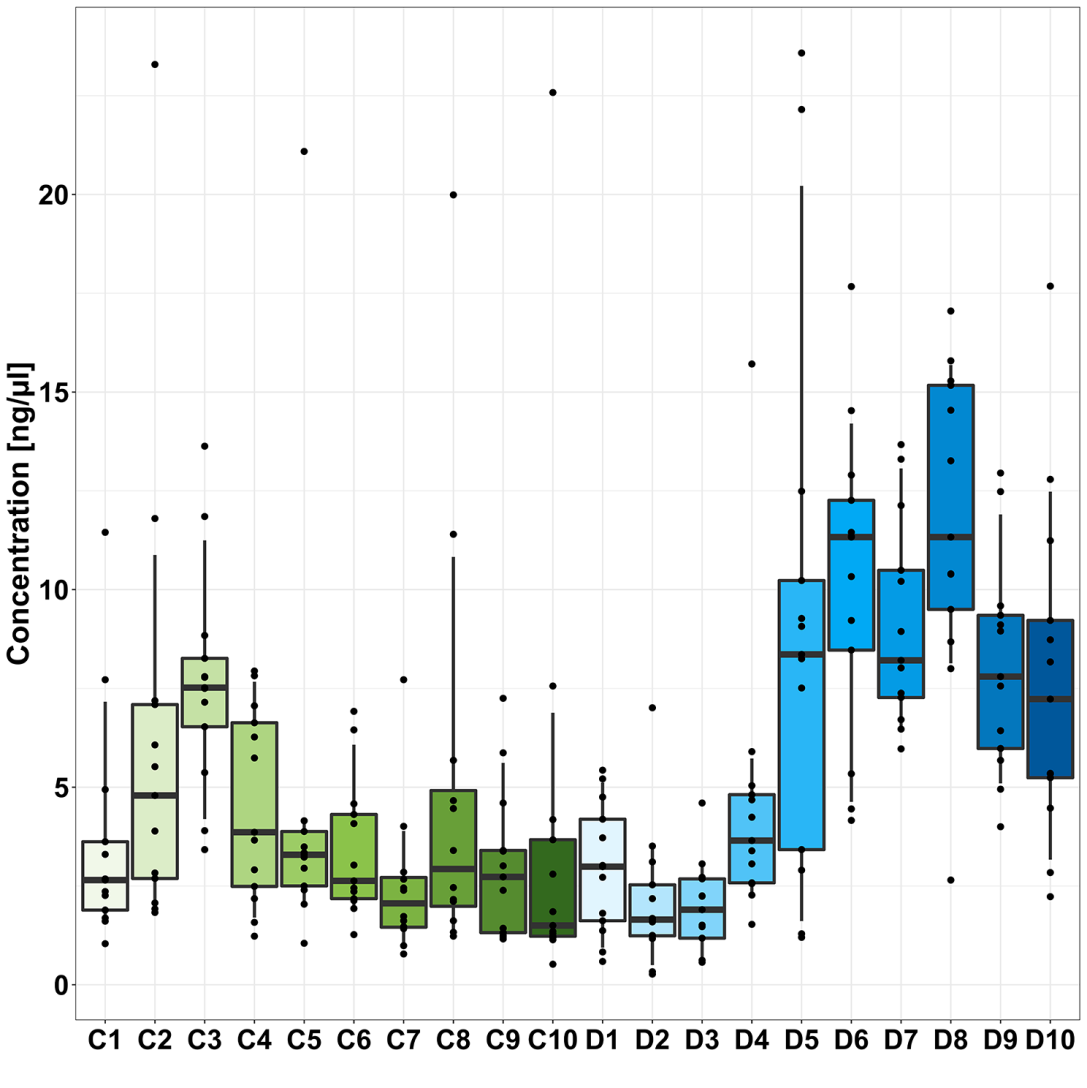
DNA concentration in T0 samples for 10 individual healthy cats (**C**) and 10 individual healthy dogs [10]

**Fig. 9:**
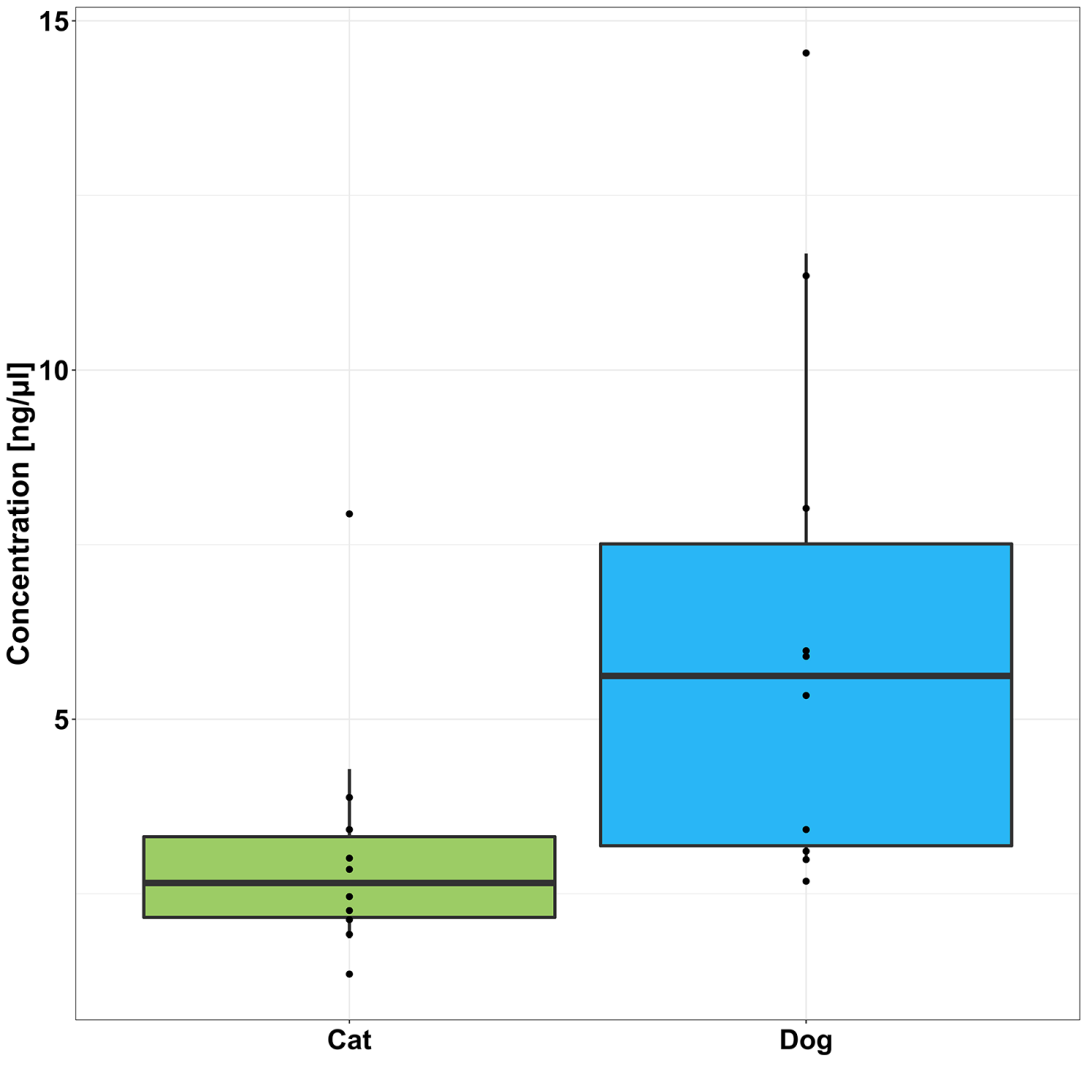
DNA concentration in core faecal samples from region ii at T0 in 10 healthy cats and 10 healthy dogs

There was individual variation in sample DNA concentration, as shown by pooled data for individuals at T0 (Fig. 8) and a significantly higher level of DNA in dog (6.3 ± 3.9 ng/µl) versus cat samples (3.1 ± 1.9 ng/µl; adjusted *p* = 0.016; Fig. 9). The wider variation in DNA concentration in dog samples was noted.

T0 R surface samples from cat faeces showed significantly higher DNA concentration when compared with T0 R core samples (5.8 ± 1.5 and 2.8 ± 2.2 ng/µl respectively; adjusted *p* = 0.044). No other significant effects of horizontal or depth sampling location on DNA concentration were seen in cats or dogs.

There was no significant change in DNA concentration across all timepoints from T0 to T6 when comparing region ii core samples (adjusted *p* = 0.99 for both cat and dog samples). DNA concentration was higher at T12 than at T0 for cat surface samples, increasing from 3.5 ± 2.1 ng/µl at T0 to 10.7 ± 7.56 ng/µl at T12 (adjusted *p* = 0.045). For cat core samples and for all dog samples, there was no significant change in DNA concentration from T0 to T12 (adjusted *p* all > 0.05).

## Discussion

The results of the study suggest that DNA concentration, alpha diversity and taxa abundance in cat and dog faeces samples are not significantly influenced by storage for up to 12 h at room temperature, horizontal area of faeces sampled, depth of sampling nor choice of two stabilisation solutions. A more global approach using Bray Curtis beta diversity metric suggests that the effect of sample collection is minor comparing to the effect of the individual. Sampling time slightly impacts dog samples diversity and not the cat samples. The location of the sampling also changes the observed diversity for the two animals, but with a low contribution to the observed variability. The two devices for dog sample collection however do not impact the beta diversity. On the PCA, the impact of sampling time and sampling location do not impair the clustering of samples according to the animal ID, highlighting the minor effect. This result is confirmed with PERMANOVA results, where the percentage of variability explained by the individual is much larger than the other R^2^ values (Fig. 7).

These results support an argument for core stool sampling in cats when studying the faecal microbiome, avoiding the possibility of surface sample contamination by litter box substrate. Lack of influence of horizontal sampling location on the outcomes was unexpected, given previous observations to suggest an effect of intestinal oxygen gradient on microbial profile [15, 16]. The proximity of the rectal end of the stool to increased oxygen gradient, and the intense dehydration of the stool at this location were expected to influence microbial profile. However, stool samples are likely to represent only the transient luminal bacteria rather than the adherent mucosal bacteria, the latter being more sensitive to the changing oxygen gradient along the colorectum [16]. When considering prolonged exposure of faeces to room temperature, the study aimed to understand how the methodology could transfer to the privately owned pet in the home environment. Whilst dog owners can collect a fresh stool from their pet during a walk, cat stools may be voided in the litter box overnight which means a delay of up to 12 h before the sample is collected and chilled. The current study suggests that storage at room temperature for up to 12 h is acceptable in terms of microbial analysis studies. However, the significant increase in alpha diversity observed in surface samples from cats taken at 12 h when compared with core samples and indications for a similar effect in dogs indicates that faecal stools exposed to room temperature for > 6 h should be sampled from the core. This was further supported by the observation of a significant increase in DNA concentration from T0 to T12 in cat surface but not core samples.

Molecular-based microbial analysis of the faeces provided valuable insights into the differences between healthy cat and dog faecal bacteria in terms of prevalence and relative abundance of certain taxa. The four most abundant phyla in cats and dogs were Bacteroidetes, Firmicutes, Proteobacteria and Actinobacteria, an observation reported in several publications [4, 7, 17–21]. In agreement with other similar studies, the current study found Proteobacteria to be more abundant in the faeces of dogs whilst Actinobacteria were more abundant in cats [22, 23]. The absence of Fusobacteria in cats was surprising, given that it has been described as one of the most prevalent phyla in faecal samples, although abundance in healthy cats is routinely reported at < 0.5% [8, 18–20]. According to a short-term (5wk) feeding study, abundance of Fusobacteria was significantly lower in cats fed dry diets (0.3%) compared with those fed wet diets (23.1%). All cats in the current study were fed dry diet with one also receiving a pouch of wet diet daily. This could account for the lack of Fusobacteria found in cats in the current study [24]. Dietary protein level is also reported to influence abundance of Fusobacteria which was reduced in growing cats fed moderate compared to high protein diets [25].

The Prevotella genus showed 100% prevalence in cats and dogs, with respective mean relative abundances of 66.7 and 49.0%. It has been suggested that the broad variety of fibres and carbohydrates typically included in commercialised pet food has promoted colonisation of the domesticated cat and dog gastrointestinal tract with genera such as Prevotella that are capable of degrading a wide range of polysaccharides [26].

The Bacteroides genus represented the second most abundant genus in cats and dogs at 14.7 and 7.8% relative abundance respectively. In dogs, the combined relative abundances of Bacteroides and Prevotella appear to be inversely related to the abundance of the Fusobacteria phylum which suggests they may occupy the same niche [9]. Evidence to support this theory is apparent in the current study where a very low relative abundance of the Fusobacteria phylum was found in dogs alongside a high Bacteroides/Prevotella abundance. Based on this, an over-representation of Bacteroides and Prevotella in cats in the current study, combining to account for 74.5% relative abundance, may help to explain the absence of Fusobacteria.

At the species level, the differences in relative abundance of *Prevotella copri* between cats and dogs was the most striking. Whilst prevalence was 100% and 82.1% in cats and dogs respectively, cats showed a significantly higher mean relative abundance (65.1%) when compared with dogs (29.5%). A recent study using whole genome shotgun metagenomic sequencing also found *P. copri* to be the most abundant species in the healthy cat gut microbiome, at 12.9% [27]. The unusually high relative abundance of *P. copri* in cats in the current study is difficult to explain. *P. copri* abundance tends to be increased in obese compared to normal bodyweight cats and dogs [27, 28], however none of the animals included in the current study were obese. The dominance of *P. copri* in cats influenced overall microbial diversity and, although not statistically significant, alpha diversity tended to be lower in cats when compared with dogs. Conversely, previous studies have indicated higher gut microbiome diversity in cats compared with dogs, although further studies are required to corroborate these findings [7, 22, 29].

The variation in microbial profiles observed across individuals was not surprising as proportions of faecal bacterial phyla in cats and dogs are known to be influenced by several factors including age, breed, diet and individual variation [7, 9, 30–33]. The current study deliberately included a non-standardised variety of animals to ensure a broad picture of the bacteria present in dog and cat faeces. However, the absence of standardisation of factors such as breed and age could have influenced inter-animal variation.

Some association patterns were observed between dietary fibre level or type and faecal microbial composition in cats and dogs (data not shown), in agreement with previous observations [34–36]. However, the small number of animals and diversity of diets in the current study necessitate additional investigation to explore this further.

There are several limitations associated with the current study that should be considered when interpreting the results. The exploratory nature of the study means that animal numbers were low and controlling for variables such as diet, age and breed of animal was not required. The results may be limited to animals fed a dry, commercial diet and the findings should be confirmed in animals fed different formats as well as in different cohorts, including privately-owned pets. The methodology used in the current study did not confirm description, function or viability of the bacterial strains identified and diversity could not be estimated using rarefaction curves as MetaPhLan3 does not have access to raw counts.

## Conclusions

This exploratory study in dogs and cats showed that, based on DNA concentration and alpha diversity, faecal samples are generally stable for up to 12 h at room temperature. The study also showed that sampling at three different regions of the faecal stool either at the surface or within the core had no significant or a few influence on the outcomes, but individual specificity is the major driver of the observed diversity. There were indications that surface samples should not be taken from faeces stored at room temperature for > 6 h. The lack of influence of stabilisation buffer allows flexible choice for further studies, which will be controlled for variables such as diet.

The results of this study will be applied to study designs for further in-house studies as well as those in privately-owned pets.

## Methods

### Animals

Ten healthy adult cats, mean age 3.8 ± 2.7y (2.2-11.1y) and 10 healthy adult dogs, mean age 3.2 ± 1.5y (1.5-5.1y) were included in the study which took place in July 2021 (see Table 1 for animal details). Animals were qualified healthy as not being affected by any disease, controlled by clinical screenings and haemato-chemical monitoring. Cats were acquired from Isoquimen company, and dogs from breeders approved by the French Research Ministry. The study was powered for detection of an effect size of 1 in terms of change from baseline. Animals were selected from a preliminary screening study, based on regular production of a stool at least 15 cm in length. Animals received appropriate routine external anti-parasitic and de-wormer treatment and were excluded if they had received any drugs in the past 14 days or antibiotics six weeks prior to the study. Animals were housed at the Royal Canin Research Centre, Aimargues, France. Housing and protocols adhered to European regulatory rules for animal welfare and the study was approved by the Royal Canin Ethics Committee, Aimargues. Animals are fed a variety of test diets (confidential nutritional composition), with no deleterious impact on the study objectives, and even better by increasing the bacterial diversity monitored. The trial was conducted as an authorised digestibility trial according to (Ref CEO90). Dogs were housed indoors individually for the duration of the study, with free access to shared outdoor runs. The inside temperature varied between 22.3 and 22.9 °C. Artificial light was provided in addition to natural light, between 06:30 and 18:30. All dogs received outdoor exercise sessions of 40–60 min per day and dog-human socialisation for 20 min per day. Cats were group-housed in social rooms with a room temperature ranging between 22.2 and 23.6 °C at the time of study. All cats received daily socialisation sessions and had free access to enclosed outdoor runs. Cats defaecated in litter boxes containing Cat’s Best® Comfort non-clumping wood-based litter substrate. Animals were fed dry, nutritionally complete and balanced diets, according to individual energy needs. One cat also received a pouch of wet food daily.

### Sample collection

A single fresh, naturally voided faecal stool was collected from each animal and stored at room temperature (20 °C) in a sealed, sterile container. Cat faeces were collected prior to the cat burying the stool in litter substrate. All faeces were scored using an adaptation of a method by Moxham 2001 [37]. Briefly, faeces are visually evaluated on a nine-point 1–5 scale, where a score of 1 is hard, dry and crumbly and a score of 5 is liquid. Faecal scores of 2-3.5 were considered acceptable for the study. A sample of approximately 1 g was taken from stored faeces at 0, 0.5, 1, 2, 3, 6 and 12 h post-collection. At T0, three horizontal regions of the faecal stool were sampled: the first emitted part (i), the middle (ii) and the last emitted part (iii), taking a sample from two depths at each location-surface and core-to give six samples at this timepoint per animal. The i and iii ends of the stool were identified using the observation that the i end is dry and the iii end is moist and soft. Surface samples of cat stools were taken from an area that had not been in contact with litter substrate. At T0.5, T1, T2, T3, T6, the sample was taken from the core of region ii for each animal and at T12, both a core and surface sample were taken from the iii region. Each sample was stored at -80 °C in DNA/RNA™ Shield (Ozyme) stabilisation solution prior to processing. To compare the use of an alternative stabilisation solution, additional aliquots from five dog samples (first emitted part (i) core, T0-T1) were placed in PERFORMABiome™ 200 stabilising solution (DNAgenotek®, Ottawa, Canada). All samples were uniquely coded and analysed blinded.

### DNA extraction

A magnetic beads-based DNA extraction was performed at Eurofins Genomics Europe (Germany), according to the manufacturer’s instructions using KingFisher™ Flex (ThermoFisher Scientific, Waltham, MA, US), with an additional sample shredding step prior to lysis. Extracted DNA was stored in 1.5 ml Eppendorf microcentrifuge tubes at − 80° C until further analysis.

### DNA preparation and sequencing

Shotgun sequencing libraries were prepared using NEB-Kit NEBNext Ultra II FS DNA Library Prep Kit for Illumina in combination with enzymatic fragmentation. Shotgun metagenomic sequencing was performed using Illumina NovaSeq 6000. At least 20 M paired-end reads/sample with sequences of approximately 150 bp in length were obtained.

### Bioinformatics analyses and statistics

Paired-end reads were assembled and low quality reads (those with Phred quality score < 15) removed. Reads were subjected to taxonomic profiling by MetaPhlAn 3.0 [38] using the ChocoPhLan database (version mpa_v30_ChocoPhLan_201901). MetaPhlAn estimates the relative abundance of microbial taxa in a metagenome using the coverage of clade-specific marker genes. The outputs were used to generate relative abundance bar plots at phylum, genus and species level.

Alpha diversity of the samples was evaluated by Shannon Index [39] and Inverse Simpson Index [40], which account for both evenness and richness of the microbial species present. Both indices are on a scale of > 0 where a higher value denotes greater diversity.

Beta diversity was assessed using the Bray Curtis distance on the relative table at the species taxonomic range. Principal component analysis (PCA) was used to visualize samples similarities on a graph. One PCA was performed with all samples from dogs and cats, and 2 others were done to represent cat and dog samples respectively. Using data from dog samples and from cat samples, 2 PERMANOVA (adonis 2 function from R package vegan 2.6-4) were performed to evaluate the variance explained by the different sample characteristics (e.g., animal ID, sampling time, Depth sampling location, Horizontal sampling location, tube).

Statistical comparisons were performed using non-parametric tests. For comparisons between two groups, Wilcoxon rank sum tests were used. Wilcoxon signed rank tests were applied in cases of paired comparisons. For comparisons between more than two groups, Kruskal-Wallis rank sum tests were used. To correct for multiple comparisons, Benjamin-Hochberg adjustments were made. Comparisons were deemed statistically significant at *p* < 0.05. Data management and statistical comparisons were carried out in R statistical software v 4.0.3.

### Electronic supplementary material

Below is the link to the electronic supplementary material.

Supplementary Material 1: Heatmap of D4 samples using the bray Curtis distance and the ward linkage method. Relative abundances of species are indicatedby the intensity of the red color.

## Data Availability

The datasets generated and/or analysed during the current study are available in the GenBank NIH at the accession number/web link: [PRJNA939514], or from the corresponding author on reasonable request.
