# Supplementary material for: Validation of method for faecal sampling in cats and dogs for faecal microbiome analysis
Source: BMC Vet Res. 2023 Dec 16;19:274. doi: 10.1186/s12917-023-03842-7 (PMC10724939; doi:10.1186/s12917-023-03842-7)
Supplement: Supplementary file 1 — Supplementary Material 1: Heatmap of D4 samples using the bray Curtis distance and the ward linkage method. Relative abundances of species are indicatedby the intensity of the red color. [file 12917_2023_3842_MOESM1_ESM.pdf]

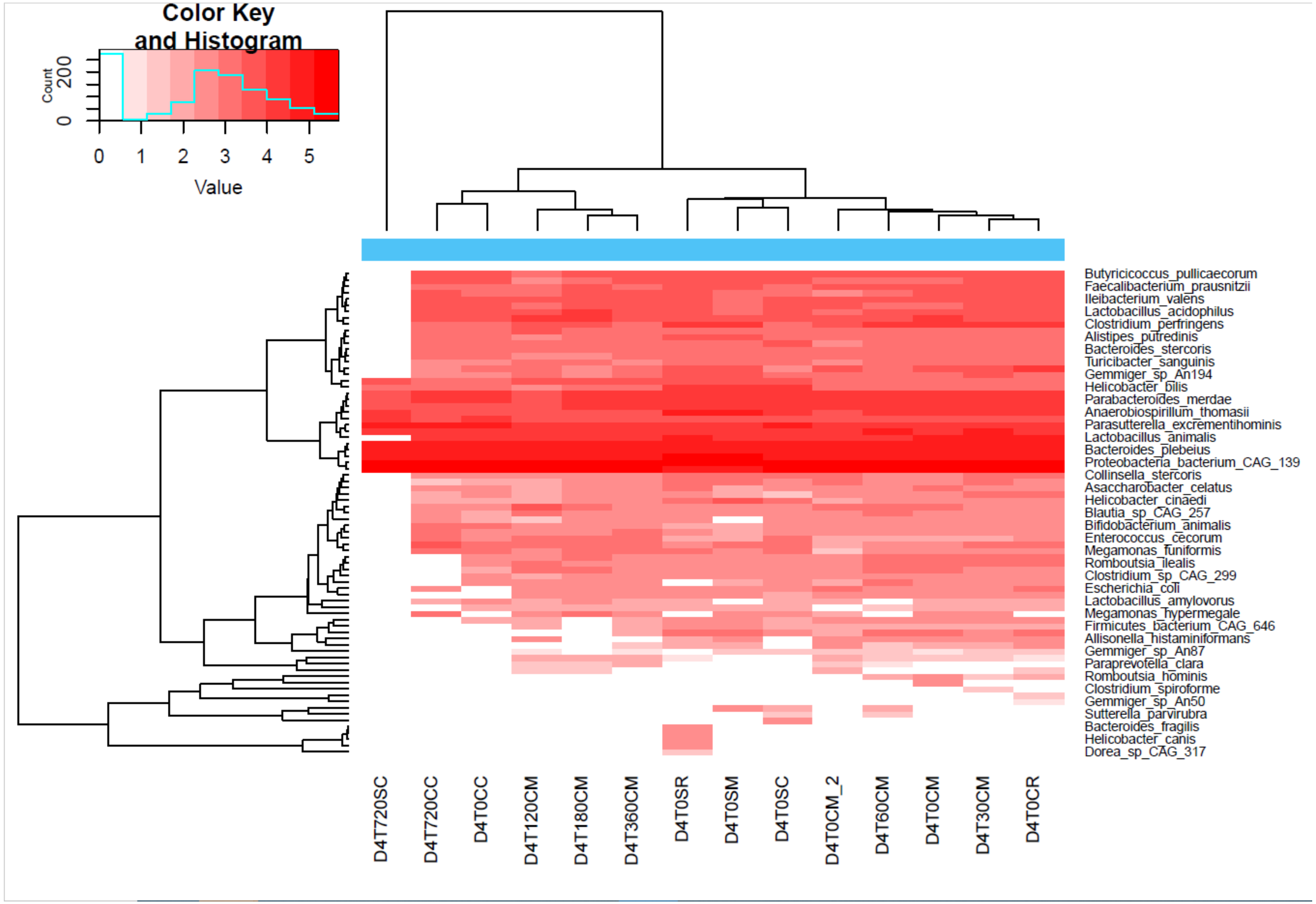

Supplementary figure 1: Heatmap of D4 samples using the bray Curtis distance and the ward linkage method. Relative abundances of species are indicated by the intensity of the red color.
